# Primary Carcinoid Tumor of the Testis: A Case Report and Review of the Literature

**DOI:** 10.1155/2018/3614387

**Published:** 2018-12-03

**Authors:** Rawad Abou Zahr, Khalil Chalhoub, Elias Mansour, Camil Chouairy, Georges Ghazal, Joe Nohra

**Affiliations:** ^1^University of Balamand, Saint George Hospital University Medical Center, Department of Urology, Beirut 1100 2807, Lebanon; ^2^University of Balamand, Faculty of Medicine, Beirut 1100 2807, Lebanon; ^3^University of Balamand, Saint George Hospital University Medical Center, Department of Pathology, Beirut 1100 2807, Lebanon

## Abstract

Carcinoid tumors usually arise in the gastrointestinal tract. Immunocytohistochemical and radiologic studies are important in detecting the primary tumor site. Primary carcinoid tumors of the testis are particularly rare with a high malignant potential warranting long-term follow-up. We present the case of a primary carcinoid tumor of the testis with long-term surveillance.

## 1. Introduction

Primary carcinoid tumor of testis is a rare type of testicular cancer accounting for less than 1% of all testicular tumors. It is associated with teratomatous components [1–3] and can occur in all ages with mean age of onset ranging between 45 and 50 [1, 3]. Among all reported cases, 12% are associated with carcinoid syndrome which is correlated with increased metastatic potential [3].

Macroscopically, carcinoid tumors are well circumscribed with calcifications seen in 10% of cases [3]. They are arranged in solid nests and acini of cells in a fibrous to hyalinized stroma. Cells consist of eosinophilic, granular cytoplasm and round nuclei with a punctate chromatin pattern. Vascular invasion is seen in 20% of testicular carcinoid tumors but is not associated with increased malignant potential [3, 4]. Cells might rarely adopt a trabecular pattern; they stain positive for substance P, serotonin, chromogranin, synaptophysin, and cytokeratin [3, 5, 6] but stain negative for OCT4, CD30, CD117, TTF-1, and CDX-2 [7]. OCT4 is a sensitive and specific marker for diagnosing seminoma, dysgerminoma, germinoma, and embryonal carcinoma [2]. CD117 and CD30 are useful in distinguishing embryonal carcinoma and seminoma, dysgerminoma, and germinoma [8]. TTF1 is a homeodomain-containing nuclear transcription protein of the NK2 homeobox (NKX2) gene family which was reported to stain positively in 53% of gastrointestinal (GI) tract carcinomas. CDX-2, caudal type homeobox 2, is used to identify tumors originating from the upper GI tract [9].

Differentiation between primary and metastatic carcinoid tumors of testis is of utmost importance for it correlates with the prognosis. The presence of teratomatous elements indicates a primary origin whereas bilateral involvement, multifocal tumors, or extratesticular extension is indicator of metastatic origin. Tumor size and the presence of carcinoid syndrome are the two important predictors of metastasis which might reach 16% roughly and hence the need for follow-up. Primary tumors have good prognosis and are usually cured by orchiectomy [2, 10].

Follow-up consists of imaging (CT or Magnetic Resonance Imaging) and urinary 5-hydroxyindoleacetic acid (5-HIAA) every three to four months in the first year. If asymptomatic and complete macroscopic resection was achieved, watchful surveillance can be adopted with yearly urinary 5-HIAA level and imaging only when level is positive [11].

We present the case of a primary carcinoid tumor of the testis with a long follow-up.

## 2. Case Presentation

A 36-year-old male patient presented with painless enlargement of his right scrotum of few weeks duration. History did not reveal any trauma or previous infections. Physical exam showed diffuse right scrotal nontender and firm mass with no palpable inguinal lymph nodes. Ultrasonography showed isoechogenic solid mass of 42x28.7 mm with two calcified foci at the apex posteriorly, the largest measuring 6.7x7.6 mm. Patient underwent right radical orchiectomy. Chest abdomen pelvis Computed Tomography (CT) scan was negative for metastasis. Patient has been followed up for 7 years and did not develop any recurrence on his yearly follow-up CT scan and urinary 5-HIAA.

Histopathologic study showed, macroscopically, parenchyma of the testis partially occupied by a solid, well circumscribed, nonencapsulated mass measuring 5x4x3.5cm with a homogenous tan gray to “creamy” color. Grossly visible areas of hemorrhage and necrosis were absent. Within the center of the mass, there was a 0.4 cm calcified nodule. Tumor is grossly separated from the tunica albuginea and from testicular hilum by a grossly unremarkable, light brown, spongy soft testicular parenchyma.

**Figure 1:**
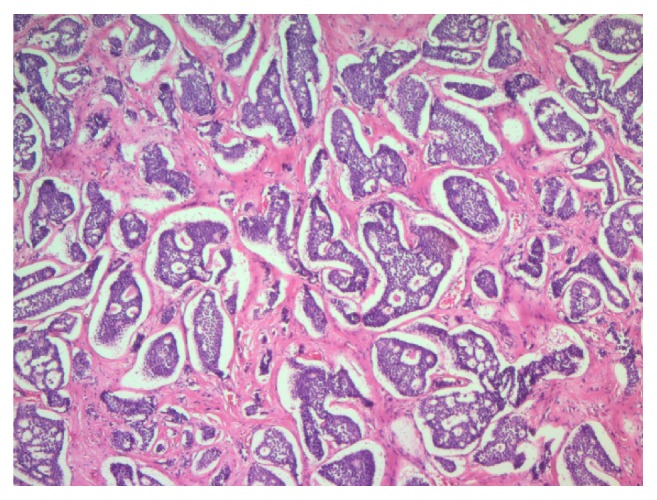
Nests and cribriform structures of variable size and shape, separated by moderate amount of fibrotic dense stroma (H&E; 50x).

**Figure 2:**
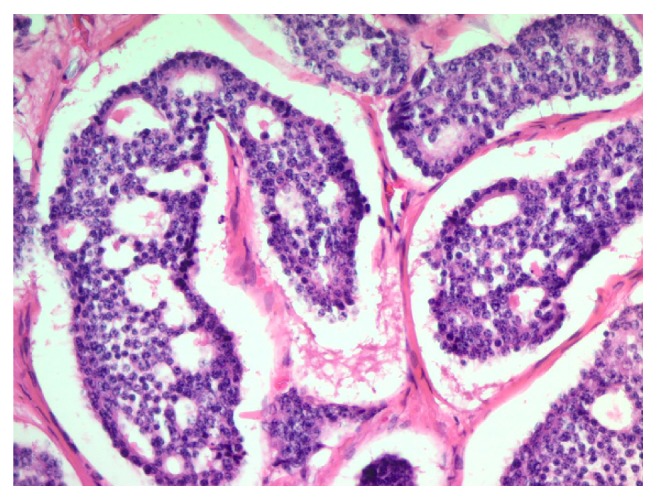
Cohesive cuboidal cells with minimal amount of cytoplasm, round uniform nuclei with regular contour, finely granular chromatin, no nucleoli, and no mitotic activity (H&E; 200x).

**Figure 3:**
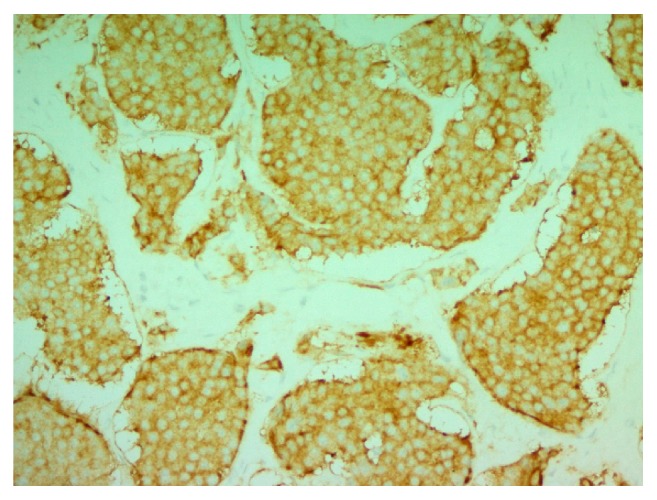
Synaptophysin immunohistochemical stain (cytoplasmic staining) (200x).

**Figure 4:**
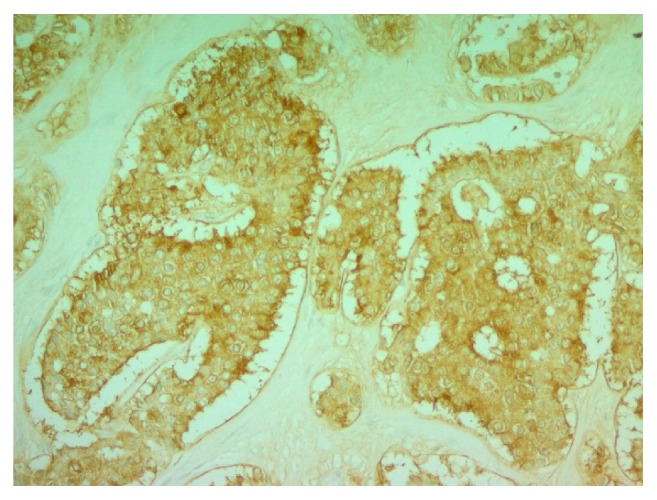
Chromogranin A immunohistochemical stain (cytoplasmic staining) (200x).

**Figure 5:**
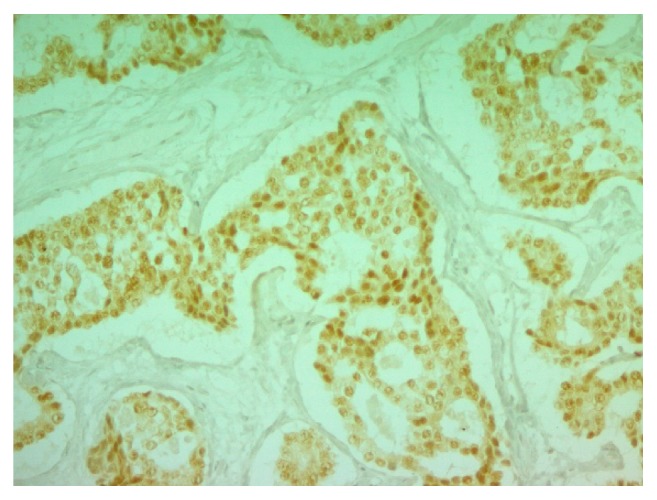
CDX2 immunohistochemical stain (nuclear staining) (200x).

Microscopically, the neoplasm is composed of cohesive, uniform, cuboidal cells with minimal cytoplasm, arranged in nests and cribriform structures of variable size and shape, separated by moderate amount of fibrotic dense stroma (Figure 1). At the periphery of the nodule, cord-like and trabecular growth patterns are noticed. Confluent as well as individual cell necrosis is absent. Neoplastic cells exhibit a uniform, round nuclei with regular contour and a granular chromatin. Prominent nucleoli are absent. Cytoplasm is pale, mild, and inconspicuous. Nuclear grooves are absent. Mitotic figures are absent to extremely rare (2 per 10 HPF) (Figure 2). Tumor cells stained positive with cytokeratin (clones AE1/AE3), synaptophysin (Figure 3), chromogranin A (Figure 4), EMA, and CDX-2 (Figure 5) but negative for CD99, TTF-1, CK7, CK20, and CEA.

## 3. Discussion

The term “carcinoid” was used to indicate a group of tumors in the small intestine arising from neuroendocrine (NE) cells having low malignant potential [12]. There is still debate concerning the carcinogenesis of primary testicular carcinoid tumors since the presence of NE cells in the testis has not been yet described with hypotheses postulating the origin to be the same precursor cell as Leydig cells, while others attributing it to a chromosomal abnormality [7, 13]. Testicular carcinoids most commonly present as a scrotal mass that can be indolent or painful in nature.

Wang et al. presented 29 primary testicular carcinoid cases and reported metastases in none of the 20 carcinoid cases with typical features and in 1 of 4 cases with atypical morphology [14]. Metastatic sites were the para-aortic lymph nodes, lungs, vertebrae, retroperitoneum, skin, and skeletal muscle [14]. Stroosma et al. reported metastases in 15.9% of cases of primary testicular lesions with no distinguishing marker for carcinoid tumors [2]. Carcinoid tumors occur in all age groups. According to Wang et al. diagnosis is based on the histopathology when there are no carcinoid symptoms or signs of metastasis [14]. 10% of carcinoid syndrome cases may metastasize to liver or lungs and manifest by erythema on upper torso and face due to the secretion of vasoactive amines, abdominal pain, diarrhea, cramps, and attacks of asthma due to the activation of vasoactive amines like serotonin [15]. The histopathologic assessment has great value in the classification of carcinoid tumors. Well-differentiated neoplastic cells have mitotic figures less than 2 per 10 HPF with mild cellular atypia. Moderately differentiated carcinoid tumors are characterized by necrosis and moderate cellular atypia and necrosis with mitotic activity more than 3 per 10 HPF and have metastatic potential [14]. Histopathologic assessment of our patient was well-differentiated carcinoid tumor.

It was reported that primary testicular carcinoids are CDX-2 negative on immunocytohistochemical staining [7]; however, a study by Lee et al. suggests that a primary testicular tumor can stain positive with CDX-2 and hence be misleading when dealing with a metastatic tumor with unknown primary which can highly be due to an occult testicular malignancy rather than of GI origin [16]. In our case, CDX-2 staining was positive; however no GI origin was found which goes in favor of occult testicular malignancy.

In a gross appearance, testicular carcinoids have been found to measure between 1 and 9.5 cm and are solid, lobulated with yellow to dark-tan and may or may not have calcifications [5]. Histologically, they are trabeculated with or without central necrosis depending on the size [16]. Grade 1 tumor cells are monomorphic with round nuclei, finely dispersed chromatin, acidophilic finely granular cytoplasm, low mitotic activity (<2/10 high power fields), and no necrosis [16]. Meanwhile higher grades show more mitoses, necrosis, and cellular pleomorphism. Grading of testicular carcinoid correlates with the clinical outcome and metastases are seen more commonly in atypical cases [4]. Histologically, our presented case revealed no atypical features.

The mainstay treatment of a localized testicular carcinoid tumor is by focal excision or radical orchiectomy, although chemotherapy and adjuvant radiotherapy have been used in metastatic cases [14]. Most carcinoid tumors are benign and have good prognosis; however, long-term prognosis is dependent on histologic grade. Wang et al. in their study showed that after a mean follow-up of 52.7 months all cases with typical features on histology had no recurrence or metastases, whereas 25% of those with atypical histology showed metastases [14]. Sasaki et al. showed metastasis 6 years after orchiectomy indicating the need for long-term follow-up [17].

## 4. Conclusion

Testicular carcinoid tumors are rare, different in presentation, and affecting all age groups. Primary carcinoid neoplasms have good prognosis but the metastatic potential exists and may be delayed; hence a long-term follow-up is warranted.

## References

[1] Berdjis C. C., Mostofi F. (1977). Carcinoid Tumors of the Testis. *Journal of Urology*.

[2] Stroosma O. B., Delaere K. P. Carcinoid tumours of the testis.

[3] Zavala-Pompa A., Ro J. Y., El-Naggar A. (1993). Primary carcinoid tumor of testis. Immunohistochemical, ultrastructural, and DNA flow cytometric study of three cases with a review of the literature. *Cancer*.

[4] Kim H. J., Cho M. Y., Park Y. N., Kie J. H. (1999). Primary carcinoid tumor of the testis: immunohistochemical, ultrastructural and DNA flow cytometric study of two cases. *Journal of Korean Medical Science*.

[5] Ordóñez N. G., Ayala A. G., Sneige N., Mackay B. (1982). Immunohistochemical Demonstration of Multiple Neurohormonal Polypeptides in a Case of Pure Testicular Carcinoid. *American Journal of Clinical Pathology*.

[6] Reyes A., Moran C. A., Suster S., Michal M., Dominguez H. (2003). Neuroendocrine Carcinomas (Carcinoid Tumor) of the Testis. *American Journal of Clinical Pathology*.

[7] Abbosh P. H., Zhang S., MacLennan G. T. (2008). Germ cell origin of testicular carcinoid tumors. *Clinical Cancer Research*.

[8] Teng L. H., Lu D. H., Xu Q. Z., Fu Y. J., Yang H., He Z. L. (2005). Expression and diagnostic significance of OCT4, CD117 and CD30 in germ cell tumors. *Zhonghua Bing Li Xue Za Zhi*.

[9] Lin F., Liu H. (2014). Immunohistochemistry in Undifferentiated Neoplasm/Tumor of Uncertain Origin. *Archives of Pathology & Laboratory Medicine*.

[10] Zavala-Pompa A., Ro J. Y., El-Naggar A. (1993). Primary carcinoid tumor of testis: immunohistochemical, ultrastructural, and DNA flow cytometric study of three cases with a review of the literature. *Cancer*.

[11] Maroun J., Kocha W., Kvols L. (2006). Guidelines for the diagnosis and management of carcinoid tumours. Part 1: The gastrointestinal tract. A statement from a Canadian National Carcinoid Expert Group. *Current Oncology*.

[12] D'Arrigo L., Costa A., Fraggetta F. (2014). Primary carcinoid tumour of the testis: A case-report. *Archivio Italiano di Urologia e Andrologia*.

[13] Mai K. T., Park P. C., Yazdi H. M., Carlier M. (2006). Leydig cell origin of testicular carcinoid tumour: immunohistochemical and electron microscopic evidence. *Histopathology*.

[14] Wang W. P., Guo C., Berney D. M. (2010). Primary carcinoid tumors of the testis: A clinicopathologic study of 29 cases. *The American Journal of Surgical Pathology*.

[15] Robertson R. G., Geiger W. J., Davis N. B. (2006). Carcinoid tumors. *American Family Physician*.

[16] Lee M. J., Vogt A. P., Hsiao W., Osunkoya A. O. (2012). CDX-2 expression in malignant germ cell tumors of the testes, intratubular germ cell neoplasia, and normal seminiferous tubules.. *Tumour biology : the journal of the International Society for Oncodevelopmental Biology and Medicine*.

[17] Ikeri N. Z., Nnaji F. C., Dawodu O., Igbokwe U., Abdulkareem F. B., Banjo A. A. (2015). Well Differentiated Neuroendocrine Carcinoma of the Testis in a Nigerian Male. *Journal of Case Reports*.

